# To divide or not to divide: revisiting liver regeneration

**DOI:** 10.1186/1747-1028-8-8

**Published:** 2013-06-20

**Authors:** Yuichiro Miyaoka, Atsushi Miyajima

**Affiliations:** 1 Laboratory of Cell Growth and Differentiation, Institute of Molecular and Cellular Biosciences, The University of Tokyo, Yayoi, Bunkyo-ku, Tokyo 113-0032, Japan; 2 Current address: Gladstone Institute of Cardiovascular Disease, University of California at San Francisco, San Francisco, CA 94158, USA

**Keywords:** Akt, Cdks, Cellular hypertrophy, Cyclins, E2F family, Hepatocyte, Liver regeneration, mTOR, Partial hepatectomy, Polyploidy

## Abstract

The liver has a remarkable capacity to regenerate. Even with surgical removal (partial hepatectomy) of 70% of liver mass, the remnant tissue grows to recover the original mass and functions. Liver regeneration after partial hepatectomy has been studied extensively since the 19th century, establishing the long-standing model that hepatocytes, which account for most of the liver weight, proliferate to recover the original mass of the liver. The basis of this model is the fact that almost all hepatocytes undergo S phase, as shown by the incorporation of radioactive nucleotides during liver regeneration. However, DNA replication does not necessarily indicate the execution of cell division, and a possible change in hepatocyte size is not considered in the model. In addition, as 15–30% of hepatocytes in adult liver are binuclear, the difference in nuclear number may affect the mode of cell division during regeneration. Thus, the traditional model seems to be oversimplified. Recently, we developed new techniques to investigate the process of liver regeneration, and revealed interesting features of hepatocytes. In this review, we first provide a historical overview of how the widely accepted model of liver regeneration was established and then discuss some overlooked observations together with our recent findings. Finally, we describe the revised model and perspectives on liver regeneration research.

## Introduction

**Figure 1:**
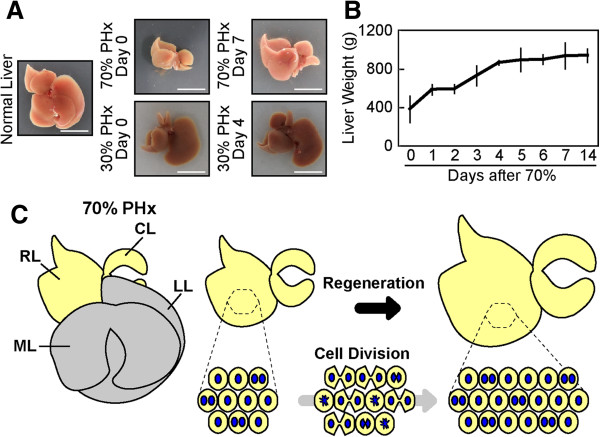
**The widely accepted model of liver regeneration.** (**A**) Liver regeneration. A normal mouse liver after 70 or 30% PHx (Day 0), and regenerated liver after 70 (Day 7) or 30% PHx (Day 4) are shown. Scale bars; 1 cm. (**B**) Liver weight change during liver regeneration after 70% PHx. The regeneration requires *circa* 14 days to recover the original liver weight. The error bars are S.D. (n = 3–7 for each day). (**C**) The currently accepted model of liver regeneration. In 70% PHx, the median lobe (ML) and the left lobe (LL) are removed, and the right lobe (RL) and caudate lobe (CL) regrow to restore the liver mass. In the traditional model, each hepatocyte is thought to divide once or twice during liver regeneration after 70% PHx. Potential alterations in size, nuclear number, and ploidy of hepatocytes are not taken into consideration.

The liver has an extraordinary capacity to regenerate from various types of injuries [1,2]. The liver consists of various cell types, including hepatocytes, biliary epithelial cells, sinusoidal endothelial cells, stellate cells, and Kupffer cells; however, hepatocytes, which carry out most of the metabolic and synthetic functions of the liver, account for about 80% of liver weight and about 70% of all liver cells [3]. In severely damaged liver with impaired hepatocyte proliferation (in this review, the term “proliferation” means an increase in cell number due to cell division), facultative liver stem/progenitor cells, which have the potential to differentiate into both hepatocytes and biliary epithelial cells, proliferate and are assumed to contribute to regeneration [2,4,5]. In contrast, regeneration after surgical resection of a portion of the liver (partial hepatectomy, PHx) does not require such stem/progenitor cells; the remnant tissue undergoes hyperplasia to recover the original liver mass within about two weeks in rodents (Figure 1A and 1B) [6-9]. In fact, this process is not a true “regeneration” like that observed in limb or heart regeneration in newts [10]. The liver does not recover the original lobular structure; rather, the remnant tissue simply increases in size (Figure 1A). Although the term, “compensatory hyperplasia” more accurately describes this phenomenon, we use “liver regeneration” in this review, as it has been used widely. The multi-lobular structure of rodent liver allows the surgical resection of a lobe of choice to achieve different degrees of liver mass loss by PHx (Figure 1A) [1]. As the resection of lobes does not induce damage to the remaining liver tissue, PHx has long been considered an excellent experimental model for tissue regeneration.

The mention of liver regeneration by Prometheus in Greek mythology indicates that ancient people had noticed the regenerative capacity of the liver. Additionally, descriptions of liver regeneration can be traced back to the 19th century when liver mass restoration with spontaneous healing of the scar was recognized after removal of a small portion of the liver [11]. In the early 20th century, it became possible to remove liver lobes by ligating blood vessels to reduce damage to the remnant liver tissue after surgery. In 1931, Higgins and Anderson carefully formulated the currently used procedure for PHx [12]. Notably, they used the term “liver restoration” instead of “liver regeneration” to distinguish clearly between compensatory hyperplasia and true tissue regeneration [12]. Since then, liver regeneration after PHx in rodents has been studied extensively for more than 80 years. Until the 1950s, liver regeneration was analyzed at mainly the tissue or cellular level by microscopic observations [13-15]. In the 1960s, the advent of electron microscopy enabled the analysis of hepatocyte ultrastructure in liver regeneration [16-19]. Almost at the same time, the epoch-making research tool of radioactive isotopes became available for biological studies. This technology was used to show that almost all hepatocytes incorporate radioactive nucleotides during liver regeneration after 70% PHx [20-25]. This landmark observation led to the establishment of the widely accepted concept that all remnant hepatocytes actively divide to recover the original cell number and liver mass (Figure 1C). This long-standing model postulated that all hepatocytes undergo roughly one or two rounds of cell division after 70% PHx [8,26,27].

Since the establishment of gene targeting technology in mice in 1989 [28-30], much effort has focused on identifying the genes required for liver regeneration. Many genes have been reported to be involved in liver regeneration after PHx [*e*.*g*., β-catenin, methionine adenosyltransferase 1A (MAT1A), oncostatin M (OSM), nuclear factor (erythroid-derived 2)-like 2 (Nrf2) and c-Met] [31-35]. Most of these studies focused on the proliferation or survival of hepatocytes in accordance with the long-standing model. Although an elegant and simple model, accumulating evidence—including our recent findings—suggest that the traditional model of liver regeneration requires revision. We discuss these observations and the proposed revised model in the following sections.

### Not all hepatocytes divide

**Figure 2:**
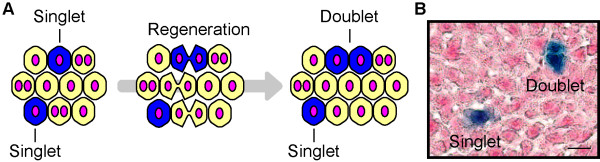
**Single hepatocyte labeling assay for evaluation of hepatocyte division.** (**A**) Schematic representation of the labeling assay. Using the optimized HTVi in Rosa26-LacZ mice, single hepatocytes (singlets) are genetically labeled by low-frequency expression of LacZ. A singlet that underwent a cell division cycle would result in a pair of neighboring labeled hepatocytes (doublet), whereas an undivided singlet would remain as a singlet. The frequency of cell division is estimated by counting the numbers of singlets and doublets. (**B**) A liver section showing a singlet and a doublet. A section of a regenerated liver with labeled hepatocytes after 70% PHx is shown. The pair of labeled hepatocytes is a doublet (upper), and the labeled hepatocyte (lower) is an example of a singlet. Scale bar; 25 μm.

The incorporation of radioactive nucleotides in hepatocytes during liver regeneration indicates that the cells entered S phase; however, this DNA replication does not necessarily mean that cell division occurred. If all hepatocytes undergo S phase and cell division after PHx, the ploidy should remain constant. However, it has long been known that hepatocyte ploidy is increased after PHx [14,36,37], suggesting that hepatocytes do not undergo conventional cell division. Previously, no convincing methods were available to investigate cell division in hepatocytes; however, we recently developed a genetic tracing method to directly assess cell division using hydrodynamic tail vein injection (HTVi) for effective delivery of plasmids into hepatocytes [38-40]. In this way, we permanently labeled hepatocytes with LacZ by transiently expressing the Cre recombinase driven by the albumin promoter in hepatocytes in Rosa26-LacZ reporter mice (Figure 2A and 2B). By randomly labeling a small fraction of single hepatocytes, the fate of LacZ^+^ hepatocytes after PHx could be precisely traced; *e*.*g*., two neighboring LacZ^+^ cells indicated that they were generated through one cell division, whereas single LacZ^+^ cells indicated that no cell division occurred (Figure 2A and 2B). Recovery of the original mass after 70% PHx occurred over the course of two weeks (Figure 1B), and we counted the number of LacZ^+^ cells during the regeneration. Surprisingly, no cell division was observed in more than 40% of hepatocytes, and the average number of cell divisions two weeks after 70% PHx was estimated as 0.7 times per hepatocyte, indicating that the number of hepatocytes increased by only 1.6-fold. Moreover, in the case of regeneration after 30% PHx (Figure 1A), hepatocytes did not undergo cell division, even though the original liver mass was recovered faster than that from 70% PHx. Interestingly, only a marginal fraction of hepatocytes entered into S phase after 30% PHx. Similar observations of infrequent S phase progression after 30% PHx were reported previously [14,41]. These observations indicate that hepatocyte proliferation alone does not account for liver regeneration after PHx.

### Hepatocytes enlarge

**Figure 3:**
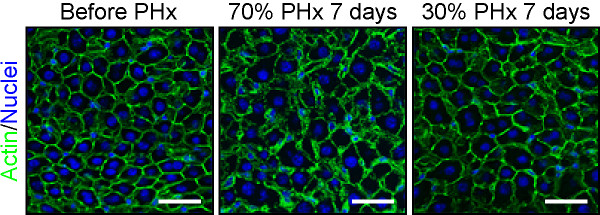
**Hypertrophy of hepatocytes after PHx.** Liver sections stained for actin (green) and nuclei (blue) are shown. The hepatocytes after 70 and 30% PHx are larger than those before PHx. Scale bars: 25 μm.

The organ size is determined not only by the cell number, but also by the size of cells that constitute the organ [42]. Because the increase in hepatocyte number (1.6-fold increase) alone could not account for the increase in the liver weight (~2.4-fold increase after 70% PHx), we investigated hepatocyte size by imaging cytometry. We found that hepatocyte size increased significantly by 1.5-fold after both 30 and 70% PHx (Figure 3) [38]. This increase in cell size alone accounts for the increased liver weight after 30% PHx, explaining the observation that hepatocytes do not divide after 30% PHx. Moreover, a combination of increased cell size and hepatocyte number account for the increase in liver weight after 70% PHx (1.5 × 1.6 = 2.4). Interestingly, increased hepatocyte size occurs as early as a few hours after 70% PHx, much earlier than their entry into the cell cycle, and peaks at 1 day after 70% PHx, suggesting that cell size increase is the first response of hepatocytes to the loss of liver mass. This very early stage of liver regeneration (0–4 hr after PHx in mice) is known as the “priming” phase, in which hepatocytes dramatically change their gene expression to prepare for regeneration [7,43]. Therefore, the change in transcriptional program seems to be responsible for the immediate hypertrophy of hepatocytes. Notably, the liver weight is almost unchanged from 1 day to 2 days after 70% PHx (Figure 1B). Because hepatocytes slightly decrease their size and start to actively enter the cell cycle from 1 day to 2 days after 70% PHx, this intervening time could be a period in which hepatocytes switch from a hypertrophic phase to a proliferative phase. The inhibition of cell cycle progression during liver regeneration has been shown to result in enlarged hepatocytes. Large hepatocytes in regenerated liver are observed in mice deficient for signal transducer and activator of transcription 3 (Stat3), S phase kinase-associated protein 2 (Skp2), separase or cyclin-dependent kinase 1 (Cdk1) [44-47]. Similarly, enlarged hepatocytes are observed in regenerating liver when the cell cycle in hepatocytes is blocked by dexamethasone [48]. However, our findings indicate that hypertrophy occurs in normal hepatocytes and precedes cell proliferation in liver regeneration. Importantly, the extent of hypertrophy is roughly the same in liver after 30 and 70% PHx, and hepatocytes do not divide after 30% PHx. Thus, hypertrophy is the first response in regeneration, and proliferation follows if hypertrophy is not sufficient to recover the original mass. In fact, Higgins and Anderson mentioned in their 1931 report that hypertrophy of hepatocytes was the first response to the removal of liver tissue [12].

It is well known that hepatocytes accumulate massive amounts of lipids and glycogen immediately after 70% PHx [49-51]. Therefore, the rapid increase in hepatocyte size is at least partly due to lipid and glycogen accumulation. However, the lipid and glycogen amount decreases to normal levels by the completion of liver regeneration, and no obvious change in hepatocyte ultrastructure is observed in regenerated liver after 70% PHx, with the exception of enlarged nuclei [38]. Although several reports have shown that hepatocytes change the size and/or number of various organelles such as mitochondria, lysosomes, endoplasmic reticulum, and ribosomes [16-19,52], more studies are necessary to reveal the nature of cellular changes in regeneration. Although the detailed mechanism of hypertrophy requires further study, the Akt-mammalian target of rapamycin (mTOR) signaling axis, which regulates the size of various cell types [53], seems to be an important pathway for hypertrophy in liver regeneration. Akt is a serine-threonine protein kinase with pleiotropic functions such as regulation of cell growth, proliferation, survival, differentiation, and cytoskeletal changes. mTOR is another serine-threonine protein kinase directly phosphorylated by Akt and plays a central role in the functions of Akt. Akt is activated immediately after 30 and 70% PHx, and forced expression of an active form of Akt in hepatocytes increases their size [[44] and our unpublished data]. Another potential key player is c-Myc, which is a transcription factor involved in cell growth and cell cycle progression. Overexpression or deletion of c-Myc in hepatocytes increases or decreases their size, respectively [54-56]. Interestingly, both Akt-mTOR and c-Myc pathways play critical roles in the enhancement of protein synthesis, indicating that upregulation of gross protein synthesis is one mechanism underlying the hypertrophy of hepatocytes [54,57]. However, the upstream molecular mechanisms that sense the loss of liver tissue to activate Akt-mTOR and c-Myc pathways remain undefined (see below).

Because hepatocytes increase their size by 1.5-fold and then proliferate after 70% PHx, the 1.5-fold increase in cell size seems to be the threshold for hepatocytes to switch their response from hypertrophy to proliferation. As discussed above, this 1.5-fold size increase is sufficient to restore a 30% loss in liver mass, and hepatocytes do not proliferate after 30% PHx. Therefore, it would be interesting to determine precisely how much liver mass must be removed to induce proliferation. The molecular trigger for hepatocyte proliferation in liver regeneration is unknown. One possible explanation is that the size of the hepatocyte itself is the sensor to drive its cell division cycle, which is considered a general mechanism for activating cell division [58]. Further studies are required to address this question.

### Hepatocytes infrequently enter M phase

Polyploidy is a characteristic feature in mammalian hepatocytes, and about 70% of adult hepatocytes in rodents are tetraploid [59]. In general, polyploid cells can arise from failed cytokinesis, mitotic slippage, cell fusion or endoreplication. Polyploid hepatocytes can be either mononuclear or binuclear. Polyploidization of hepatocytes is initiated in postnatal liver growth by incomplete cytokinesis, that produces binuclear polyploid hepatocytes, endoreplication that produces mononuclear polyploid hepatocytes, or both that produce binuclear polyploid hepatocytes [37,59,60]. Insulin signaling has been implicated in the polyploidization and binucleation at the weaning stage, as discussed below [37,60,61].

While it has long been known that ploidy of hepatocytes increases after PHx [14,36,37,62], its mechanism remains unknown. Although a majority of hepatocytes undergo S phase in regenerating liver after 70% PHx, not all hepatocytes undergo cell division, resulting in an increase in ploidy. We noticed that the ratio of hepatocytes that were positive for phosphorylated histone H3 (an M phase marker) to those that were positive for Ki67 (a G1 to M phase marker) in liver regeneration is much lower than that in postnatal liver, where hepatocytes actively undergo cell division [38]. These results suggest that M phase progression is compromised in liver regeneration.

M phase promoting factor (MPF), composed of cyclin B and Cdk1, regulates entry into M phase [63,64], and MPF must be activated for cell transition from G2 to M phase. Cdk1 is phosphorylated at three amino acid residues (Thr-14, Tyr-15, and Thr-161) in the inactive form of MPF, whereas Cdk1 dephosphorylation at Thr-14 and Tyr-15 by cell division cycle 25 (Cdc25) activates MPF [65]. We found that the phosphorylation level of Cdk1 at Tyr-15 was much higher in regenerating liver compared to postnatal liver. Therefore, the lower activity of MPF in regenerating liver could be a cause of the infrequent entry into M phase. In fact, MPF activity is dispensable for liver regeneration; hepatocytes increase their size to regenerate liver after 70% PHx without cell division, even in the absence of Cdk1 [47].

In contrast to the G2 to M phase transition, the G1 to S phase transition is driven mainly by cyclin D/A2 and Cdk2 in normal cell division and endoreplication [66]. Therefore, it is intriguing to compare the activity of Cdk2 in liver regeneration with that in liver development. As hepatocytes increase their ploidy in both postnatal liver development and regeneration, the cell cycle regulators driving the G1 to S phase transition seem to dominate those driving the G2 to M phase transition in mature hepatocytes. Although the exact molecular mechanism that blocks the entry of hepatocytes into M phase in regenerating liver remains elusive, a crucial role of the E2F family transcription factors has been reported recently [67,68]. The E2F family consists of E2F transcription activators and transcription repressors and regulates cell cycle progression. Using mouse genetic models, these studies clearly showed that atypical E2F repressors E2F7 and E2F8 inhibit the completion of cell division to enhance polyploidy and binucleation in hepatocytes both in liver development and regeneration, whereas the canonical activator E2F1 counteracts their activities. These E2Fs differentially control the transcription of cell cycle regulators to either enhance or inhibit the G2 to M phase transition.

### Cell division of binuclear hepatocytes to produce mononuclear cells

Binucleation is another interesting feature in adult hepatocytes that begins from the neonatal liver [60]. It has long been known that the number of binuclear hepatocytes decreases during liver regeneration after 70% PHx, as assessed by microscopic observations and manual counting [13-15,69-71]. Weaning increases the amount of circulating insulin to activate Akt signaling, which induces incomplete cytokinesis to generate binuclear hepatocytes during liver maturation [37,60,61]. In contrast, even though Akt is activated by PHx, the number of binuclear hepatocytes decreases in regenerating liver, suggesting that Akt has different functions in liver maturation and regeneration. Indeed, Akt signaling induces hypertrophy of hepatocytes in liver regeneration (our unpublished data). The different responses to Akt may be due to the molecular targets of the Akt signaling pathway differing according to the cellular context. mTOR is a major downstream molecule of Akt that functions in induction of hypertrophy. Because the E2F family transcription factors regulate the progression of M phase [67,68], it is tempting to speculate a link between Akt signaling and E2Fs in binucleation. Indeed, it is already known that this link exits in other cell types [72,73].

**Figure 4:**
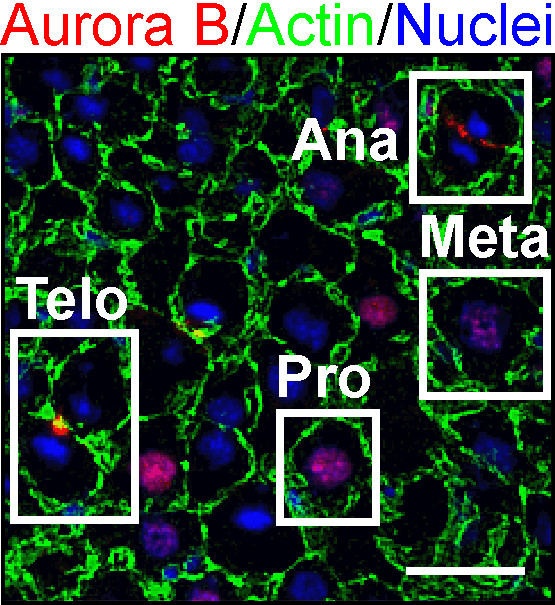
**Hepatocytes in different stages of M phase distinguished by intracellular localization of Aurora B.** A section of liver stained for Aurora B (red), actin (green), and nuclei (blue) is shown. The dynamic change in intracellular localization of Aurora B and nuclear morphology allow discrimination of hepatocytes in prophase (Pro), prometaphase/metaphase (Meta), anaphase (Ana), and telophase (Telo). Scale bar: 25 μm.

To elucidate the cellular basis underlying the reduction in nuclear number in liver regeneration, we investigated the behaviors of mononuclear and binuclear hepatocytes during liver regeneration using the genetic tracing method and observation of intracellular localization of Aurora B [38]. The intracellular localization of Aurora B differs among the M phase steps [74]; therefore, we could distinguish hepatocytes in prophase, prometaphase/metaphase, anaphase and telophase (Figure 4). We found that 32% of hepatocytes in prophase were binuclear, compared to only 1.9% of cells in prometaphase/metaphase. Furthermore, all hepatocytes in anaphase showed splitting of the two nuclei to their two poles, and 93% of pairs of daughter hepatocytes in telophase consisted of two mononuclear cells. Therefore, almost all cell divisions seemed to produce daughter mononuclear cells irrespective of the nuclear number of mother hepatocytes. Based on these observations, we speculate that mononuclear mother cells follow the normal cell division cycle, whereas binuclear mother cells gather their chromosomes at the center of the cells and split two nuclei to two daughter cells again. Consistently, the genetic tracing method showed that almost all pairs of neighboring daughter hepatocytes produced by cell division were pairs of two mononuclear hepatocytes [38]. Interestingly, this mode of cell division of binuclear hepatocytes was predicted from microscopic observations of hepatocytes in the early studies of liver regeneration [13,70]. Moreover, the same mode of cell division was later reported *in vitro*[75]. Our results reinforce this older prediction and suggest that this mode of cell division also occurs in binuclear hepatocytes *in vivo*. Although division of hepatocytes with multipolar spindles has been reported *in vitro*[76], further studies are required to address whether it also occurs *in vivo*.

Binucleation is generally considered a sign of terminal differentiation in both hepatocytes and cardiomyocytes [77,78]; however, in contrast to binuclear hepatocytes, binuclear cardiomyocytes do not divide. Mononuclear and binuclear cardiomyocytes seem to have distinct functions, and it has been proposed that only mononuclear cardiomyocytes maintain their proliferative potential to serve as stem or progenitor cells in heart muscle [79,80]. It is unclear whether hepatocytes with different numbers of nuclei have different functions. This issue is discussed further below.

### A revised model of liver regeneration

**Figure 5:**
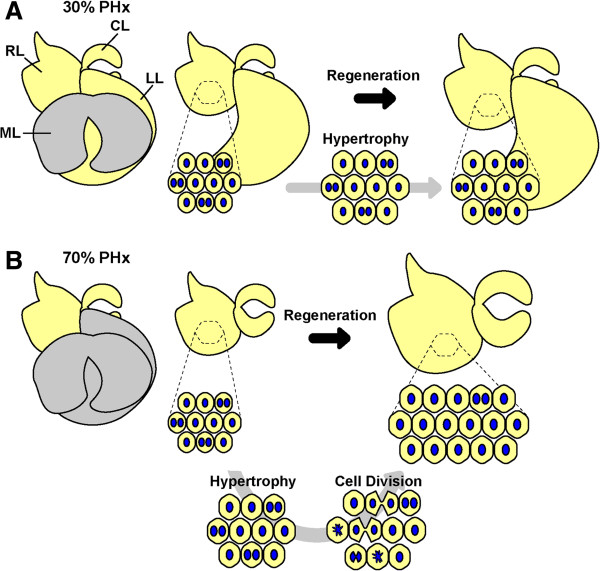
**The revised model of liver regeneration.** (**A**) Liver regeneration after 30% PHx. The median lobe (ML) is removed and the left lobe (LL), right lobe (RL), and caudate lobe (CL) regrow. During this process, all hepatocytes enlarge, but some enter S phase and divide only rarely. As a result, hepatocytes slightly increase their ploidy, but do not change their nuclear number. (**B**) Liver regeneration after 70% PHx. All hepatocytes increase their size and then enter the cell cycle to undergo S phase. Some cells execute cell division to produce mainly mononuclear daughter hepatocytes, irrespective of nuclear number of mother hepatocytes. However, not all hepatocytes divide. Liver recovers its lost mass by a combination of hypertrophy and proliferation. As a result, hepatocytes increase their ploidy, but decrease their nuclear number.

Based on our findings, together with previous observations, we have proposed a revised model of liver regeneration [38]. Upon 30% PHx, the liver recovers its original mass by increasing the size of hepatocytes, but neither the cell number nor the nuclear number of hepatocytes changes. Furthermore, because only a small fraction of hepatocytes undergo S phase, their ploidy is not altered significantly (Figure 5A and Table 1). In contrast, when 70% of liver is removed, hypertrophy of hepatocytes occurs in a few hours after PHx, followed by cell proliferation. Almost all hepatocytes enter into S phase, but only about half undergoes cell division to increase their numbers. During proliferation, binuclear hepatocytes seem to preferentially undergo unconventional cell division, in which chromosomes from two nuclei are split into two nuclei to produce two mononuclear daughter hepatocytes. As a result, the nuclear number decreases, whereas ploidy increases (Figure 5B and Table 1). Although there are still some other possibilities to be considered, such as hepatocyte fusion and/or nuclear fusion during liver regeneration, we believe that this revised model represents the characteristic behavior of hepatocytes during liver regeneration and is more accurate than the traditional model.

**Table 1:** **Cellular properties of hepatocytes in regenerated liver after 30 and 70**% **PHx**, **compared to normal liver**

	**Cell number**	**Cell size**	**Nuclear number**	**Ploidy**
**30% PHx**	Decreased	Increased	Unchanged	Marginally increased
**70% PHx**	Decreased	Increased	Decreased	Increased

### Cellular robustness of hepatocytes

Adult hepatocytes can be binuclear, polyploid and even aneuploid under normal conditions [76,81]. Furthermore, cell number, cell size, nuclear number and ploidy of hepatocytes are significantly different in normal liver and regenerated liver after 30% or 70% PHx (Table 1). Despite these differences, liver seems to function almost equally in different conditions, which raises an intriguing question whether such differences in the cellular properties affect hepatocytes. One study using transcriptomic analysis showed that hepatocytes with different ploidy were basically indistinguishable [82]; however, another study indicated that polyploid cells were more resistant to stressful conditions [83]. Hepatocytes with different ploidy were shown to be equally susceptible to interferon-γ (IFN-γ)-induced apoptosis [84]. Proliferation of polyploid hepatocytes was compromised and they exhibited more characteristics of senescence [85]. No consensus has been reached on the functional differences in hepatocytes of different ploidy or number of nuclei. The volume of hepatocytes is basically proportional to their ploidy, which is often the case with other cell types [42,84,86,87]. However, we noticed that hepatocytes increase their size without increasing their DNA content after 30% PHx [38], suggesting that hypertrophy without increased ploidy allows hepatocytes to function properly. Naturally occurring aneuploidy is another feature of hepatocytes, which seems to arise from inaccurate chromosome segregation [76,81]. Aneuploidy is often associated with genetic disorders and is observed in various cancers [88,89]. However, aneuploidy does not seem to be tumorigenic in hepatocytes, and it may even provide genetic diversity in hepatocytes to perform different functions [81].

In addition to these characteristic features exhibited by wild-type mice, genetically modified mice show rather extreme phenotypes of hepatocytes. Mutant mice with impaired cell cycle progression showed a fully functional liver with extraordinarily enlarged hepatocytes after 70% PHx [44-47]. A loss of E2F7 and E2F8 reduced ploidy or nuclear number but did not affect hepatocyte function and regeneration after several liver injuries including PHx [67,68]. Furthermore, hepatocytes were resistant to DNA damage caused by a lack of telomeric repeat binding factor 2 (TRF2). In the absence of TRF2, liver regenerated by increasing the size and ploidy of hepatocytes and was fully functional after 70% PHx [90]. These observations indicate collectively that hepatocytes have a “cellular robustness” which allows them to perform their functions in a variety of settings; these differ in terms of cell size, ploidy or nuclear number. The observed extreme plasticity in ploidy of hepatocytes supports their robustness [76]. It is tempting to speculate that the cellular robustness of hepatocytes is one reason why of mammalian organs, only the liver has such a marked regenerative capacity.

### Size control of organs

Liver regeneration serves as an excellent model for regulation of organ size. Generally, differences in organ size among animals reflect differences in cell number rather than cell size [91,92]. Limb regeneration in amphibians also depends on an increase in cell number to fully recover the original tissues. However, the size and number of hepatocytes in liver regeneration after PHx contribute differentially to the recovery of liver mass. It has been suggested that removal of one kidney induces the enlargement of the other by increasing the size of kidney cells [93]. Moreover, physiological and pathological cardiac hyperplasia is induced by hypertrophy of cardiomyocytes [94,95]. Therefore, cellular hypertrophy could be a general mechanism for increasing organ size. Hippo/mammalian Ste-20 like kinase (Mst)1/2-Yorkie/Yes-associated protein (YAP) signaling plays an indispensable role in the regulation of organ size [96-99]. In normal adult hepatocytes, Mst1/2 kinase (the mammalian homologue of *Drosophila* Hippo) phosphorylates and inactivates YAP (the mammalian homologue of *Drosophila* Yorkie), which is a transcription activator that induces cell proliferation and suppresses apoptosis. Transgenic expression of human YAP in mouse hepatocytes drastically increased the liver size [96]. This regulation of organ size by Hippo/Mst1/2-Yorkie/YAP signaling might be due mainly to the control of proliferation and apoptosis in cells [100]. However, Hippo/Mst1/2-Yorkie/YAP signaling affects cell size by tuning Akt-mTOR signaling via miRNA [101], demonstrating that cell size plays a critical role in organ size regulation by Hippo/Mst1/2-Yorkie/YAP signaling as well.

What senses and regulates liver size is a fundamental question. The decreased number of hepatocytes and increased size of hepatocytes in regenerated liver suggests that liver size is determined by the total mass of hepatocytes. PHx drastically changes the blood flow into the liver. This increased blood flow generates shear stress that induces nitric oxide production, triggering regeneration [102-106]. In addition, the amount of bile acid in the blood might serve as a mechanism of monitoring the size of liver because it reflects the total mass of the hepatocytes [107,108]. Additionally, because the liver serves as a major reservoir of glycogen, the blood glucose level reflects the liver mass and so might also be a sensor. Consistent with this hypothesis, it has long been known that rodents become hypoglycemic after PHx, and supplementation of glucose inhibits liver regeneration [109-111]. This inhibitory effect of glucose is suggested to be mediated by p21 [112]. Other factors in the regulation of liver size might be cytokines and serum proteins secreted from hepatocytes. A key contributing feature of these factors is that they must reflect the total mass of hepatocytes, but not the number or size of individual hepatocytes. Although these factors may sense the liver size, the mechanism of initiating and promoting regenerative responses remains unknown. Furthermore, liver regeneration must terminate when the liver recovers its original mass. Several molecules have been suggested to be involved in the termination of liver regeneration including transforming growth factor-β (TGF-β), a mitoinhibitory cytokine for hepatocytes [113]; extracellular matrix, which might inhibit proliferation of hepatocytes via integrin-linked kinase (ILK) and glypican 3 [114-116]; and peroxisome proliferator-activated receptor-γ (PPAR-γ) a mitoinhibitory transcription factor for hepatocytes [117]. However, the termination of liver regeneration has been inadequately studied compared to the initiation process. Recent transcriptome analyses of termination may shed light on its underlying molecular mechanisms [118,119]. A future challenge is to elucidate the molecular links between the sensors of liver size, the factors that regulate the hypertrophic and proliferative responses of hepatocytes, and the termination process of liver regeneration that acts to maintain the appropriate liver size.

## Conclusions

Although liver regeneration has been studied extensively, many important fundamental mechanisms remain undefined such as the mechanisms of cellular hypertrophy, cell division, nuclear division, ploidy changes and organ size control. Liver regeneration after PHx provides an excellent experimental system to tackle such basic biological questions. Understanding the mechanisms underlying liver regeneration is clinically important because hepatectomy is a practical treatment for liver tumors, and liver transplantation is an important therapeutic option in patients with severe liver diseases. Understanding the mechanism of liver regeneration will lead to the development of promising therapeutic strategies.

## Competing interests

The authors declare that they have no competing interests.

## Authors’ contributions

YM and AM wrote the manuscript. YM prepared the figures and table. Both authors read and approved the final manuscript.
